# MRI in Glioma Immunotherapy: Evidence, Pitfalls, and Perspectives

**DOI:** 10.1155/2017/5813951

**Published:** 2017-04-20

**Authors:** Domenico Aquino, Andrea Gioppo, Gaetano Finocchiaro, Maria Grazia Bruzzone, Valeria Cuccarini

**Affiliations:** ^1^Neuroradiology Unit, Fondazione IRCCS Istituto Neurologico “Carlo Besta”, Milan, Italy; ^2^Postgraduate School in Radiodiagnostics, Università degli Studi di Milano, Milan, Italy; ^3^Molecular Neuro-Oncology Unit, Fondazione IRCCS Istituto Neurologico “Carlo Besta”, Milan, Italy

## Abstract

Pseudophenomena, that is, imaging alterations due to therapy rather than tumor evolution, have an important impact on the management of glioma patients and the results of clinical trials. RANO (response assessment in neurooncology) criteria, including conventional MRI (cMRI), addressed the issues of pseudoprogression after radiotherapy and concomitant chemotherapy and pseudoresponse during antiangiogenic therapy of glioblastomas (GBM) and other gliomas. The development of cancer immunotherapy forced the identification of further relevant response criteria, summarized by the iRANO working group in 2015. In spite of this, the unequivocal definition of glioma progression by cMRI remains difficult particularly in the setting of immunotherapy approaches provided by checkpoint inhibitors and dendritic cells. Advanced MRI (aMRI) may in principle address this unmet clinical need. Here, we discuss the potential contribution of different aMRI techniques and their indications and pitfalls in relation to biological and imaging features of glioma and immune system interactions.

## 1. Introduction

Glioblastoma multiforme (GBM) is the most common primary brain tumor in adults [1] and carries a grim prognosis.

Infiltrative nature of diffuse gliomas makes it difficult to eliminate microscopic disease despite macroscopic gross total resection. Recurrence of GBM is inevitable, and the median overall survival (OS) time of GBM patients receiving the standard treatment, which consists of maximal safe resection followed by radiation and adjuvant temozolomide, is about 14–16 months [2, 3]. At recurrence, no standard approach has been established (further surgery, reirradiation, chemotherapy, and antiangiogenic therapy) and despite advances in treatment for GBM, the survival of patients has not significantly improved over the past two decades.

The central nervous system (CNS) has been traditionally considered an immune-privileged system; however, it has been proved that immune cells can cross the blood-brain barrier (BBB) to gain access to the brain parenchyma and can leave the CNS to reach the cervical lymph nodes. Considering that the immune system has access to the brain and that GBM expresses multiple tumor antigens that can be targeted by immunotherapeutic approaches, the development of immunotherapy has gained considerable interest over the last decade [4].

Converging data indicate that cancer epitopes can be recognized by the immune system and therefore an immune reaction can be mounted to erase or block tumor growth. Resistant tumor clones, grown under immune pressure, create an immune suppressive environment that leads to the formation of relevant tumor. These general observations also apply to brain tumors. Cancer immunotherapy strategies are aimed at reverting such immune suppression [5].

Novel immunotherapeutic strategies being investigated to treat glioblastoma can be broadly divided into three major classes: active immunotherapy, adoptive immunotherapy, and immunomodulatory strategies [6]. They include vaccination therapy targeted against specific tumor antigens or whole tumor lysate, adoptive cellular therapy with cytotoxic T lymphocytes, chimeric antigen receptors and bispecific T-cell engaging antibodies to bypass major histocompatibility complex restriction, aptamer therapy allowing a more efficient target delivery, and checkpoint blockade to release the tumor-mediated inhibition of the immune system. Within active immunotherapy, to enhance the immunogenicity of GBM, two approaches are used nowadays: (a) peptide immunotherapy where the target is a cancer-specific antigen like EGFRvIII (epidermal growth factor receptor) and (b) dendritic cell immunotherapy where dendritic cells act as antigen-presenting cells and can be pulsed with autologous tumor lysate or peptides from cytomegalovirus that is present in GBM but not in normal brain [7]. Checkpoint inhibitors that have been used to treat advanced tumors with durable remission in some cases are now in clinical trials in GBM patients: they facilitate effective antitumoral immune response as they suppress coinhibitory pathways activated by neoplasms to suppress T-cell responses against tumor cells [8].

Initial data show prolonged OS (23 to 38 months) in GBM patients treated by vaccines [9]. Upcoming clinical trials' results will clarify the efficacy of different cancer immunotherapy approaches, in particular using checkpoint inhibitors. Due to the heterogeneity of glioblastoma, multiple treatment strategies of immunotherapy, in addition with conventional therapy, will be more likely to succeed.

Efficacy of therapy is assessed by clinical examination and magnetic resonance imaging (MRI). Pseudoprogression, that is, imaging features suggesting tumor progression that is not confirmed subsequently, occurs in up to 30% of patients within three months after radiochemotherapy [10–12]. Thus, considering pseudoprogression as true tumor progression (and conversely) could lead to an inappropriate change in therapy and errors in assessing the efficacy of novel treatments [13]. Pseudoprogression during immunotherapy seems to occur more often, and its timeframe remains to be defined, potentially differing by the class of immunotherapy given.

To address these issues, the iRANO (immunotherapy response assessment in neurooncology) committee redefined the response assessment criteria for patients with neuro-oncological malignancies undergoing immunotherapy: the “limbo” window when radiologic worsening does not suggest immunotherapy suspension has been widened to six months, after which true progression, if detected, should be backdated [14].

Conventional MRI (cMRI) has limitations in differentiating tumor progression/recurrence and immunotherapy responses [15]. Advanced MRI (aMRI) may allow a deeper understanding of tumor structure and biology. Unlike contrast enhancement, increased perfusion may be independent of BBB integrity and defines tumor neoangiogenesis [16]. On diffusion-weighted imaging (DWI), the apparent diffusion coefficient (ADC) inversely correlates to tumor cellularity [17]. MR spectroscopy (MRS) provides information about metabolites within tumoral and perilesional tissue [18].

Amino acid PET (mainly with methionine and fluoroethyltyrosine) has been used to enlighten the greater metabolic activity of malignant tumoral tissue compared to radionecrosis and might also help in differentiating progression from treatment-related alterations during immunotherapy [19–22]. Such facilities are restricted to a limited number of specialized centers. A review on amino acid PET, however, is beyond the aim of this review.

Evidence that aMRI techniques can differentiate pseudoprogression and tumor recurrence has been reported in radiotherapy and chemotherapy, and promising data suggest they may differentiate at early-stage responder and nonresponder patients to immunotherapy. The purpose of this review is to summarize current research on MRI assessment for patients undergoing immunotherapy with a major focus on aMRI parameters.

## 2. Magnetic Resonance Imaging (MRI)

### 2.1. Conventional MRI (cMRI)

Several criteria have been proposed in the last two decades to assess response to therapy in gliomas: the standard method is based on contrast-enhancing images in T1 and on hyperintensity in T2 or FLAIR (fluid-attenuated inversion recovery) sequences. Nevertheless, enhancement on T1 reflects nonspecific impairment of the BBB, a reduction or lack of enhancement can be due to tumor shrinkage but also to antiangiogenic therapy, due to vascular normalization besides tumor infiltration (pseudoresponse). On the other hand, in pseudoprogression, an early, subacute reaction to treatment (e.g., radiotherapy) is associated with contrast enhancement, edema, and possible mass effect, and sometimes, associated clinical symptoms initially suggest tumor progression but subsequently resolve without any further treatment and can be associated to longer survival [23]. Furthermore, T2 and FLAIR hyperintensity can be associated to tumor infiltration, but also to edema, ischemia, gliosis, demyelination, inflammation, or postactinic alteration. In particular, inflammation might mimic radiological features of tumor progression with increased enhancement including new lesions and edema.

Pseudoprogression is generally not associated with clinical deterioration in radiochemotherapy [13] but can be linked to increased edema and clinical symptoms during immunotherapy. Since effective immune responses might need time to develop, early imaging might reflect true progressive disease and only later be followed by delayed response. Notably, previous experience in melanomas showed that while tumor regression is often low (about 10%), many patients could have prolonged periods of disease stabilization [24].

Volume of enhancement lacks to differentiate between progressive disease and pseudoprogression. Moreover, the pattern of enhancement in pseudoprogression is not specific and can be nonhomogeneous, mimicking GBM, nodular, “cottony,” and sometimes quite intense as in “flare” inflammatory phenomena also observed after local intracerebral/intratumoral immunotherapies [25].

RANO (response assessment in neurooncology) criteria, including cMRI were published in 2010 to address the issues of pseudoprogression after radiochemotherapy and/or pseudoresponse during antiangiogenic therapy [26]. To overcome limitations of previous criteria, T2/FLAIR assessment of the lesions was included, especially in patients treated by antiangiogenic antibodies like bevacizumab; pseudoprogression after radiotherapy was considered if recurrence was present in the radiated field within 12 weeks after the completion of radiotherapy and required a repeated scan after 4 weeks to confirm or exclude progression; corticosteroid use and clinical status were also considered. Current RANO criteria are based on two-dimensional measurements on MRI. However, there is an ongoing debate on to whether volumetric measurements would be more accurate in defining tumor evolution over time, and the inclusion of such measurements as secondary study endpoints is encouraged [19].

Pseudoprogression can be more frequent after immunotherapy. The precise mechanism of pseudoprogression, occurring in up to 30% of patients with glioblastoma after radiochemotherapy, is poorly understood [12, 27]. In some immunotherapy cases, histopathology showed infiltration of CD8+ lymphocytes, but not mitotically active tumor cells [28]. Effective immune response might need time to evolve, and early imaging might reflect true progressive disease; on the other hand, inflammatory response in areas of macroscopic or microscopic infiltrative tumor might mimic radiological features of tumor progression with increased enhancement and edema.

In 2009, the increased interest in evaluating immunotherapies led to the development of immune-related response criteria (irRC) [24]: these guidelines considered that *inflammatory responses* may imply transient enlargement of the tumor or the appearance of new lesions thus complicating the assessment of tumor progression and recommended that since new lesions do not necessarily indicate progression, patients with enlarging lesions should repeat the scan 4 weeks later.

**Figure 1:**
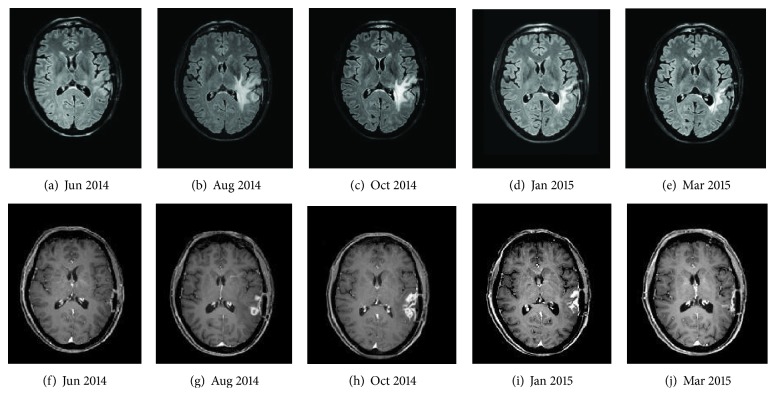
FLAIR (a–e) and contrast-enhanced T1-weighted images (f–j): postsurgical (a, f), increasing edema (b, c), enhancement (g, h) and subsequent reduction (d, e, i) of both, and remission of the enhancing lesion (j) in the course of immunotherapy with dendritic cell vaccine.

The iRANO committee, integrating guidance for progressive imaging findings from the irRC with RANO criteria, redefined the response assessment criteria for patients with brain tumors undergoing immunotherapy providing novel iRANO criteria [14]: in patients with early findings suggesting progression (i.e., ≥25% increase in the sum of biperpendicular diameters of enhancing tissue, development of new lesions, or substantial worsening in T2/FLAIR) within the first 6 months of immunotherapy regimen without substantial neurological decline, therapy should be continued and confirmation of radiographic progression by follow-up imaging should be sought 3 months after the initial radiographic evidence of progressive disease (Table 1, Figure 1).

### 2.2. Delayed-Contrast MRI: TRAMs (Treatment Response Assessment Maps)

In recent years, Zach et al. proposed a new method to distinguish active tumor and treatment-induced effects [29]. The method implies the acquisition of two high-resolution 3D T1-weighted sequences in the same MR session, 3–5 and 60–75 minutes after the injection of the contrast medium and in the subsequent subtraction of early from late sequences. The map obtained is then color-coded to differently represent areas in which contrast accumulates during time (red-coded) and regions in which contrast is rapidly cleared from the tissue (blue-coded). Histological validation allowed to identify blue regions as the active tumor regions and red areas as treatment-induced regions in which vessel lumen resulted disrupted and contrast tended to accumulate. The maps obtained are defined as treatment response assessment maps (TRAMs). Semiautomated calculation of the volume of each component can be performed and longitudinally monitored.

Different from other methodologies, TRAMs are not user-dependent, less acquisition-dependent (i.e., they only need good-quality 3D T1 sequences), and relatively simple to be acquired.

The inconveniences are that patient has to wait longer outside the scanner and that timing of postcontrast acquisitions is quite critical. The choice of the first time point is important because right after contrast injection, the gadolinium signal rises fast and the signal has to be high when the images are acquired in order to be sensitive to tumor regions (blue). On the other hand, this acquisition time point has to be early enough not to lose sensitivity to treatment effects (red). The closer to the maximal peak value, the larger is the difference between early and delayed signal and, consequently, sensitivity. For these reasons, 3–5 minutes should be used and, due to the fast changes in signal intensity, it is important to fix this time point for each patient follow-up. The choice of the second time point is mainly affected by the time the tumor takes to clear gadolinium from the tissue. Inter- and intratumor variability in clearance times exist, but after 1 hour, the signal changes slowly; therefore, the second time point can be flexible (to allow for a practical clinical application in a busy radiology department) between 1 and 1.45 hours postinjection.

#### 2.2.1. Response Assessment

**Figure 2:**
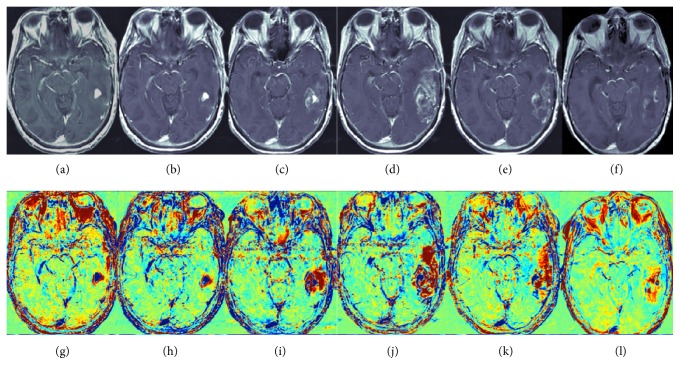
Contrast-enhanced T1-weighted images (a–f) and the calculated TRAM postchemoradiation (g–l) (images were acquired 0.7, 2.5, 4, 6, 7, and 8 months postchemoradiation). Temporary enlargement of enhancing lesion (c-d) is shown; as it can be seen, the red volume growth rate was prevalent above the blue volume (i-j), favoring pseudoprogression over progression. Pseudoprogression was later confirmed by the decrease in all volumes 7 and 8 months postchemoradiation (e-f, k-l). Modified from [110] with permission.

TRAMs have been used in radiochemotherapy and antiangiogenic therapy allowing discrimination between tumor and treatment-related effects (Figure 2) with sensitivity 100% and positive predictive value 92%, demonstrating different TRAM patterns on pretreatment and early treatment stage in responder versus nonresponder patients [29, 30]. In these studies, TRAMs showed higher accuracy than cerebral blood volume (CBV) in PWI (see Section 2.3 for details).

#### 2.2.2. Immunotherapy

The rationale for applying TRAM analysis to immunotherapy lies on the differentiation between tumor and immune cells: preliminary data showed different components in enhancing lesions during immunotherapy with dendritic cells, with prevalence of “blue” regions in early progressive cases. However, longer follow-up in responder versus nonresponder patients is needed to understand if TRAMs can define immune-mediated pseudoprogression as they do in postradiotherapy follow-up.

### 2.3. Perfusion-Weighted Imaging (PWI)

Three different MRI techniques have been developed to study brain microvascular hemodynamics. Two are based on the injection of gadolinium-based contrast medium, dynamic susceptibility contrast (DSC) and dynamic contrast enhanced (DCE), and the other, arterial spin labeling (ASL), uses blood as an internal contrast medium.

#### 2.3.1. Dynamic Susceptibility Contrast (DSC)‐MRI Perfusion

DSC [31] aims to study the hemodynamic characteristics of the brain microvascular network after the injection of a paramagnetic contrast medium and the contemporary dynamic acquisition of the brain volume. The estimation is obtained indirectly, starting from the signal intensity change caused by the passage of the contrast agent. The injection of a highly concentrated bolus is rapid (about 4-5 ml/sec), in order to cause an appreciable signal decrease. The sequence is an echo-planar spin (SE)/gradient-echo (GRE), and DSC acquisition lasts 1-2 minutes.

After the acquisition, in the postprocessing step, a signal-time curve is extracted from every voxel of the brain volume. The curve is composed of a first baseline, prior to contrast arrival, a sharp peak corresponding to contrast bolus arrival and a final portion that represents contrast recirculation. Starting from the signal, the concentration of the contrast medium during time is mathematically obtained in every voxel. From every concentration-time curve obtained, four semiquantitative parameters can be derived: (a) CBV, defined as the ratio between the blood volume passed in a region and that entering that region [31], is an indirect measurement of the regional capillary bed density and an indicator of neoangiogenesis [32]; (b) cerebral blood flow (CBF), representing microvascular blood flow rate in that region, is estimated by the concentration-time curve of the contrast entering the region to be examined called arterial input function (AIF), obtained by placing a region of interest (ROI) on one of the major arteries of the brain; (c) mean transit time (MTT), the time necessary to the contrast medium to pass through the area under examination; and (d) time to peak (TTP), the time necessary to reach the maximum contrast concentration.

CBV has found applications in the study of brain gliomas for tumor grading [16, 33, 34], in the distinction between recurrence and radionecrosis [35] and in the prediction of clinical outcome and response [36, 37].

BBB disruption is a frequent condition in brain gliomas. In this condition, the contrast molecules can leak from vascular space and reach the parenchyma. This results in an unwanted T1-weighted effect leading to under- or overestimation of CBV that can be overcome by the injection of a prebolus of contrast to saturate T1.

The principal limitations of DSC are related to the sensitivity of the sequence to BBB disruption or susceptibility artifacts at natural interfaces (e.g., trabecular bone, paranasal sinuses, skull base, and sella), generally heavier at high field strengths.

#### 2.3.2. Dynamic Contrast Enhanced (DCE)‐MRI Perfusion

This technique uses gadolinium to characterize the BBB and estimate its damage [38]. Different from DSC, the sequence acquired is T1-weighted (typically a 3D spoiled gradient-echo sequence) since, due to BBB disruption, contrast medium crosses the endothelial wall accumulating in the extravascular tissue causing T1 enhancement. DCE takes advantage of the signal intensity increase: dynamics is slower (acquisition time is 6–10 min), due to the time for contrast medium to be washed in and out.

The first step is the conversion of enhancing signal-time curves into concentration-time curves. From the concentration-time curves, a first not specific index can be derived: the initial area under the curve (iAUC). Comparable to CBV, this index could give indication about contrast leakage. Higher iAUC values generally correspond to more malignant conditions, where vessel permeability is high. This index however includes multiple information such as flow and permeability.

In order to have more detailed and specific information about BBB damage, the tissue in each voxel is represented by a multicompartment model composed of vascular, extravascular, and intracellular space. Using pharmacokinetic models [39], it is possible to estimate quantitative parameters to characterize the vascular damage: (1) *K*_trans_, the most frequently used metric in tumor assessment representing the rate of transfer between plasma and extravascular tissue; (2) *K*_ep_, the transfer rate between extravascular tissue and plasma; (3) *V*_e_, the extracellular volume, inversely correlated to high cellularity and mitotic activity [40]; and (4) *V*_p_, the fractional plasma volume [41].

With DCE, it is possible to extract quantitative values on BBB damage and intra- and extravascular volumes. Their estimation however is limited by water exchange phenomena, by the choice of the AIF, and by the robustness of the fitting procedure. Moreover, DCE uses a T1-weighted sequence that, different from DSC, is not affected by susceptibility artifacts. A 3T scanner or higher is preferable.

#### 2.3.3. Arterial Spin Labeling (ASL)

This technique does not use any external contrast medium: the contrast is the blood entering the brain that, magnetically labeled, can be used to estimate the CBF [42]. The first applications of ASL used a long radiofrequency (RF) pulse simultaneously with a selection gradient to adiabatically invert the spins of the feeding arteries (continuous ASL, CASL). Recently, rather than a long RF pulse, a train of short RF pulses combined with a strong gradient has been used (pulsed CASL, pCASL) [43].

The main limitations are related to the intrinsically poor signal-to-noise ratio, due to the low fraction of free-water spins and to their relaxation. For this reason, the sequence generally used is echo-planar, allowing the acquisition and the averaging of multiple volumes.

#### 2.3.4. Response Assessment

DSC has been used to evaluate early responses [44–46] to antiangiogenic therapy [47] and chemoradiation [48]. Parametric response maps (PRMs) of CBV [49] have been recently compared to a classical ROI-based approach in the identification of recurrent GBMs responsive to bevacizumab and irinotecan (antiangiogenics) [50]. In GBM, DSC has been used in combination with ASL and DTI (diffusion tensor imaging, see Section 2.4 for details) to discriminate between progression and pseudoprogression in radiochemotherapy [51, 52]. Pseudoprogression is typically characterized by reduction of CBV, relative peak height, and increase of signal intensity recovery [53]. CBV < 1.75 was predictive of pseudoprogression versus true progression [54].

DCE has been used for differential diagnosis [55] and tumor grading [56, 57]. Prognostic value of *K*_trans_ has been particularly investigated, showing a correlation between high baseline levels and poor PFS and OS [58]. *K*_trans_ and CBV, interestingly, did not co-correlate with prognosis. In patients with recurrent GBM, mean values of *V*_p_ and *K*_trans_ were significantly reduced in the pseudoprogression but not in tumor progression [59].

ASL has recently found application in the study of brain tumors [60–64]. A retrospective analysis [65] demonstrated higher sensibility of ASL-CBF than cMRI to identify tumor progression. ASL in combination with DSC was used to discriminate pseudoprogression and real progression in 117 newly diagnosed GBM treated with chemoradiation [51]. ASL produced eight (12.9%) more accurate results than DSC alone. In progressive and stable GBMs studied by using DSC, DCE, and ASL, perfusional values (CBV, CBF, ASL-CBF, and *K*_trans_) were higher in progressive lesions: the most accurate technique was DSC (CBV and CBF) [66].

#### 2.3.5. Immunotherapy

DSC perfusion has been studied during immunotherapy confirming that elevation of CBV in a region with contrast enhancement supports the diagnosis of malignant tumor [67, 68].

In eight patients treated with dendritic cell immunotherapy [67], the maximum normalized lesional CBV resulted highest in progressing tumors, intermediate in preprogressing lesions, and lowest in stable cases. Although a clear correlation between CBV and pseudoprogression was not achieved due to the small number of cases, the authors support maximum CBV as a potential radiological marker to differentiate between immunotherapy-induced inflammatory response and GBM recurrence.

Interestingly, a mismatch between enhancing volumes and high CBV volumes has been described in 11/79 examinations in three of six immune-treated patients: the region with elevated CBV was never larger than the region with contrast enhancement. Histopathological evaluation in two cases showed malignant cells with numerous proliferating vessels with thrombosis or ruptures [68]. On the other hand, areas of enhancing tissue without hyperperfusion could be a sign of reactive, inflammatory changes due to immune-mediated BBB impairment: this hypothesis is supported by histopathology in immune-treated brain metastases of melanoma, showing reactive astrocytosis and scattered inflammatory and microglial cells surrounding isolated clusters of tumor cells [69].

DCE at 7T field strength has been used in immunotherapy studies in rat models of GBM: increased Ve was found in tumors responding to treatment due to tumor cell death and reduced proliferation, as indicated by the decreased growth index on histology. On the contrary, progressive lesions exhibited the greatest growth index and Ve was decreased with a tendency to reduce transvascular transport (*K*_trans_) [70].

As vessel permeability can be affected by inflammation, endothelial junctions become less tight, *K*_trans_ higher. Clinical studies are ongoing to evaluate permeability in immunotherapy and to associate DCE parameters to pseudoprogression or true progression and to clinical outcome.

ASL has not yet been used in immunotherapy follow-up and would be an option in patients with renal failure and severe allergy to contrast agents, also limiting the potential risk of chronic contrast accumulation [71].

### 2.4. Diffusion MRI

#### 2.4.1. Diffusion-Weighted Imaging (DWI)

Brownian motion of water molecules inside the tissues brings water to diffuse in different brain compartments. This motion can be detected using a modified version of a spin-echo sequence that includes two strong gradients (diffusion gradients) [72]. The strength and geometrical characteristics of these gradients are summarized by a scalar value, the *b* value. The aim of these gradients is to magnetically label the spins in some directions of the space so as to catch their motion and reconstruct tissue architecture. Being dynamic phenomena, the sequence used is an echo-planar sequence that lasts few minutes and needs the acquisition of two volumes at different *b* values, generally 0 and 1000 s/mm^2^. In DWI acquisition, one coefficient only is calculated, the ADC, a measure of the amount of water diffusivity inside the tissues. ADC is inversely proportional to the cellularity concentration [73]: vasogenic edema results in higher ADC values, with increased extracellular water content, whereas cell swelling produces low ADC values. ADC has been largely used to study brain gliomas [74] and also stroke and neurodegenerative disorders. Different from PWI, DWI measures are not affected by user-dependent parameters and ADC values are consistent if scanner parameters are controlled and remain the same. Increased ADC levels could indicate a first response to radiochemotherapy [75].

#### 2.4.2. Diffusion Tensor Imaging (DTI)

Different from DWI that only gives information about the amount of water displacement, diffusion tensor imaging allows to infer water-motion directionality [76]. With DTI, it is possible to characterize and reconstruct the main pathways of white matter fibers. The base sequence used is the same but is repeated in at least six noncollinear directions of the space [77]. For this reason, a DTI acquisition generally lasts about 10–12 min. By putting together the values obtained in each direction, it is possible to estimate the three preferential directions along which water moves in that tissue. From these directions, some quantitative parameters can be calculated: (a) fractional anisotropy (FA), an index describing directionality of the white matter fibers in the voxel; the higher the FA value (maximum corresponds to 1, minimum to 0), the higher is the voxel directionality. If the value is low, the movement of water spins could be retained isotropic. (b) Mean diffusivity (MD), similar to ADC, represents the water diffusivity degree. (c) Axial diffusivity (AD), the value of the principal eigenvalue, that is, the degree of movement along the principal direction, strictly related to axonal damage. (d) Radial diffusivity (RD) represents water diffusivity in a direction transversal to the principal and related to the myelinization degree. These values are calculated point by point and maps are constructed.

The role of DTI metrics in tumoral characterization is debated. Some studies found that the tumoral core is characterized by low MD and high FA values [78], whereas peritumoral edema shows high MD and low FA values identifying FA as a marker of tumor infiltration [79]. In rat gliomas, low FA values were found in the center of the lesion, high FA values in the peritumoral rim, and high MD, RD, and AD in the perilesional edema [80]. Even if DTI has been used for tumor grading [78, 81, 82], there is no uniform consensus about the role of its metrics in glioma characterization. This is probably due to limited standardization of the results and validation with histopathology [83].

Starting from the DTI metrics and from the directional information extracted in each voxel, it is possible to reconstruct three-dimensionally the pathways of white matter fibers. This technique, called tractography, is widely used in association with functional MRI before tumor resection [84].

#### 2.4.3. Response Assessment

DWI has been widely performed to characterize brain gliomas and monitor radiochemotherapy or antiangiogenic therapy [85]. Functional diffusion maps (fDMs) [75] and histogram analysis need [86] to be highlighted. These techniques, which apply ADC maps to characterize the heterogeneity of therapy response, have been used to evaluate the efficacy of cytotoxic and antiangiogenic therapies [85, 87, 88].

Progressive disease in high-grade gliomas, different from pseudoprogression, seems characterized by low ADC values due to hypercellularity [76, 77, 79]. ADC studies were often conducted in conjunction with other MRI modalities to improve the characterization of glioma heterogeneity [89–91].

DTI has increasingly been performed to study high-grade gliomas; histogram analysis and fDMs can provide early evidence of low-grade glioma modifications during chemotherapy with respect to cMRI (i.e., RANO criteria [14]), but only few studies have used it to discriminate progression and pseudoprogression [92–94]. DTI after radiochemotherapy shows elevated levels of FA in tumor progression compared to pseudoprogressing enhancing lesions; longitudinal DTI without segmentation was also proposed [94].

#### 2.4.4. Immunotherapy

During immunotherapy, an inflammatory reaction is expected, carrying edema and reduced tumor cell density on one side but immune cell accumulation and hypercellularity on the other side as in other brain inflammatory diseases such as encephalitis or abscesses that exhibit lower ADC values than normal brain [95, 96].

In a pilot study on eight patients treated with dendritic cell immunotherapy, minimum ADC levels were lower in enhancing lesions at progression [67]. Furthermore, ADC levels within nonenhancing, FLAIR hyperintense regions were lower in preprogressive than in stable lesions. The parameter might be predictive of response: even if the small number of cases (three progressive diseases and five stable diseases, pseudoprogression not specified) did not allow the characterization of treatment-induced effects, the authors support ADC as a potential radiological marker to differentiate immunotherapy-induced inflammatory response and GBM progression.

Serial parametric response mapping of ADC in 21 children carrying pontine glioma treated by peptide-based vaccination following radiation therapy showed fractional decreased ADC in the four patients experiencing pseudoprogression [97].

Very recently, in a retrospective analysis of 10 recurrent GBM patients [98], intermediate ADC (IADC) volumes of interest (VOI) were able to discriminate the five patients with clinical benefit (i.e., without unequivocal clinical radiologic or histopathologic evidence of glioma progression for at least five months since trial onset) from the others. IADC VOI represented voxels within the FLAIR VOI having ADC in the range of highly cellular tumors (0.7–1.1 × 10^−3^ mm^2^/s). IADC VOI started to decrease in patients with clinical benefit three months on average after immunotherapy onset while in other patients, the value continued to increase.

DTI has been proposed in the follow-up of gliomas with treatments other than immunotherapy [90] and could be performed in longitudinal follow-up of selected patients. The main limit of this technique during immunotherapy may be the frequent presence of substantial edema due to inflammation.

### 2.5. Magnetic Resonance Spectroscopy (MRS)

MRS aims to study brain metabolism identifying and quantifying some relevant metabolites in a specific region. The water proton is most commonly used, as it is easy to implement in most of the scanners. Unlike MRI, which uses the two-dimensional signals to derive images of the brain, MRS uses a monodimensional ^1^H signal to estimate relative metabolite concentrations. Two are the principal MRS modalities: (a) chemical shift imaging (CSI) that gives a spatial distribution of the metabolites taking at the same time spectra deriving from multiple brain voxels (a grid) and (b) single voxel spectroscopy (SVS) that only acquires spectra from a little portion (VOI) of the brain. Both SVS and CSI do not cover the entire brain volume.

The most common metabolites investigated by MRS are (a) N-acetyl aspartate (NAA), a neuronal marker decreasing when neuronal integrity is affected; (b) choline (Cho), a marker of increased cellular turnover usually elevated in tumors and inflammatory processes; and (c) creatine (Cr), which gives a measure of energy storage. In brain tumors, NAA results generally decreased and Cho increased, probably due to the membrane turnover. Other metabolites whose concentration generally changes in brain tumors are lactate, due to the anaerobic glycolysis; lipids, probably because of membrane disruption and necrosis; and myoinositol, associated to “crowding” of glial cells. Most recently, tumor characterization and therapeutic monitoring benefited from the possibility to study 2-hydroxyglutarate (2HG), an oncometabolite accumulating in tumors carrying isocitrate dehydrogenase (IDH) gene mutations [99–101]. Data indicate that IDH1 mutations are immunologically targetable [102]. In a translational model, vaccination with peptides encompassing the mutation in mice transgenic for human major histocompatibility complex (MHC) classes I and II caused MHC class-II-restricted antitumor immune responses based on CD4+ T-cells [102, 103].

Absolute quantitative MRS gives the concentration of the metabolites in a given voxel. Ratios of metabolite concentrations and metabolic maps (i.e., colorimetric maps reporting the single metabolite or ratio values in every voxel of the CSI grid), can be obtained.

#### 2.5.1. Response Assessment

MRS has been used for glioma diagnosis, grading, and response monitoring [104, 105]. It may discriminate recurrent gliomas and radiation necrosis, but the most accurate parameters result in normalized Cho/NAA and Cho/Cr ratios with 88% and 83% sensitivity and 86% and 83% specificity, respectively [66, 106]. Other studies [107], however, showed low levels of NAA and high levels of Cho in both tumor progression and pseudoprogression [90, 91, 107], in particular at early phases after radiation [108].

#### 2.5.2. Immunotherapy

**Figure 3:**
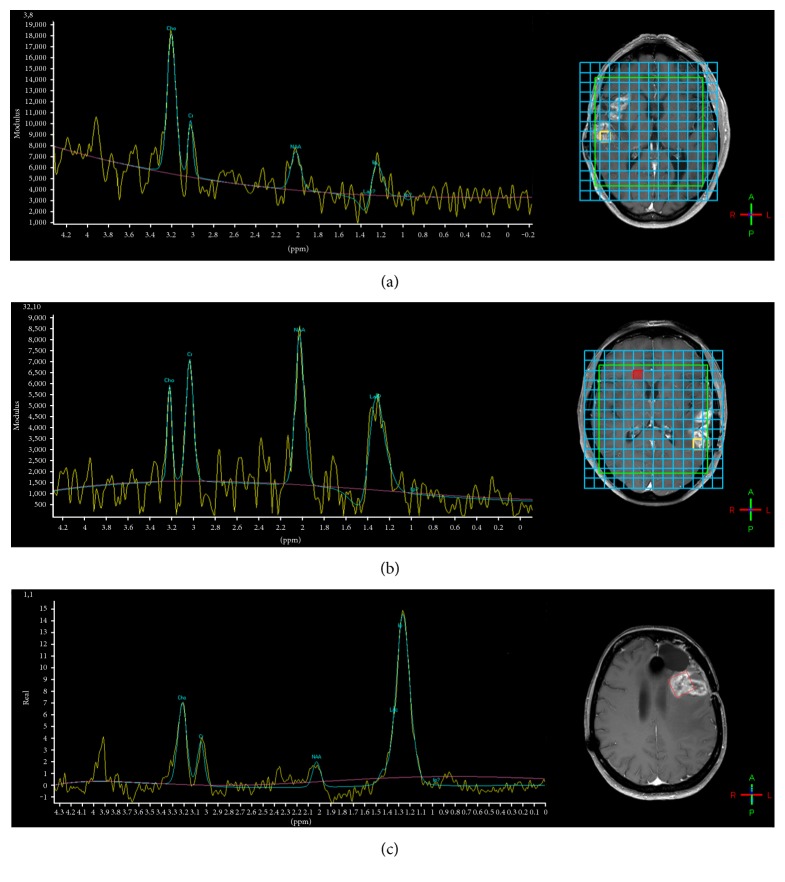
MRS during immunotherapy, after surgery, and radiotherapy plus temozolomide. Left, spectra; right, voxel positioned within enhancing lesions. (a) High Cho and low NAA with minimum lipids in recurrent glioma. (b) Preserved Cho and NAA levels with evident though not prevalent lipid peak in pseudoprogression (the same case is shown in Figure 1—time point Oct 2014). (c) Prominent peak of lipids and lower peaks of Cho, Cr, and NAA but with high Cho/Cr and Cho/NAA ratios in mixed scenario with glioma recurrence and radionecrosis.

GBM is usually characterized by high concentration of Cho, decreased Cr and NAA, and presence of lipids in necrotic areas. Because of the inter- and intraindividual heterogeneity of high-grade gliomas, metabolite concentrations can vary considerably. MRS can detect the presence of high Cho levels (and Cho/Cr or Cho/NAA ratios) within enhancing and perifocal tissue thus enlightening the presence of glioma: specificity is high but mixed scenarios with coexistence of glioma and treatment alterations are frequent (Figure 3). Spectroscopic maps from multivoxel acquisitions are useful to monitor Cho distribution and concentration in the altered field of interest in longitudinal follow-up.

MRS findings were reported in two GBM patients after multimodal treatment with surgery, radiation, intralesional immunotherapy (IL-4 toxin), and chemotherapy: pseudoprogression was observed with extensive and increasing enhancement which nearly completely regressed after four to six months. In both patients, MRS did not show increased Cho within the enhancing areas [109].

A “harmonic” reduction of Cho, Cr, and NAA in the presence of lipids is usually associated with radionecrosis while lipids in the presence of elevated Cho/Cr ratio and low NAA suggest the presence of high-grade glioma. In immunotherapy, lipids have been described as substrate of NK T-cells [110] and the presence of a lipidic peak might be associated to a better immunotherapy response. Thus, MRS correlations with immunological findings can be interesting, given the relevance of NK responses in recurrent GBM receiving immunotherapy with dendritic cells [111].

### 2.6. Susceptibility-Weighted Imaging (SWI)

Susceptibility-weighted imaging (SWI) is a tool for high-resolution imaging of the vasculature. The technique relies on the phase signal of a T2^∗^ sequence to amplify contrast between veins and brain tissue based on their susceptibility differences. The sequence is a fully flow compensated, long echo, 3D gradient-recalled-echo (GRE) pulse sequence, lasting about 5–7 minutes. Magnitude and phase volumes derived by sequence acquisition are combined together to produce a new image particularly sensitive to iron, calcium, ferritin, and venous blood [112]. Postcontrast SWI modification in relation to precontrast SWI indicates that signal alterations in tumors are not the result of calcification or subacute hemorrhage. Moreover, SWI has enlightened heterogeneity in enhancing lesions. The main advantages of SWI are high reproducibility and the gain of information on macro- and microvasculature without contrast medium.

SWI has a role in the evaluation of the vascular organization of brain gliomas and of neoangiogenesis that rapidly produces small, tortuous, and immature vessels leading to microbleedings and surrounding edema and also in the identification of tumor calcifications.

#### 2.6.1. Response Assessment

SWI has been used for tumor grading [113–116] and differential diagnosis [117], analyzing the presence of the so-called “intratumoral susceptibility signals” (ITSS), defined as “low signal intensity and fine linear or dot-like structures, with or without conglomeration, seen within the tumor” [118] often present in GBM and absent in lymphomas [117]. Because hypointense signal on SWI images has been shown to reflect both vascularity and vascular integrity, this technique has a potential as a predictive marker and in assessing treatment effects and response to antiangiogenic treatments or radiotherapy [119–121]. Additionally, on SWI sequences, significant higher concentrations of gadolinium accumulate at the border of the tumoral invasion zone.

#### 2.6.2. Immunotherapy

SWI data in immunotherapy have not yet been reported.

This technique, with contrast medium, might have a potential in immunotherapy response assessment as SWI features are a surrogate of vascularity and are more pronounced on lesional borders, where enhancement is frequently prominent in immune-treated tumors or inflammatory diseases. SWI might be considered to differentiate enhancing GBM from areas rich in immune cells, given that ITSS can be found in high-grade gliomas and the development of new ITTS suggests recurrent or progressive disease, features that are absent in lymphomas (i.e., hypercellular and lymphocytic tumors) [117] and not described in inflammatory conditions. Moreover, edema does not significantly interfere with SWI images.

## 3. Concluding Remarks and Perspectives

In spite of the improvement determined by RANO criteria first and iRANO subsequently, the imaging definition of the actual dynamics of glioma and immune cell interactions and their impact on patient survival during checkpoint or DC immunotherapy remains unsatisfactory.

**Figure 4:**
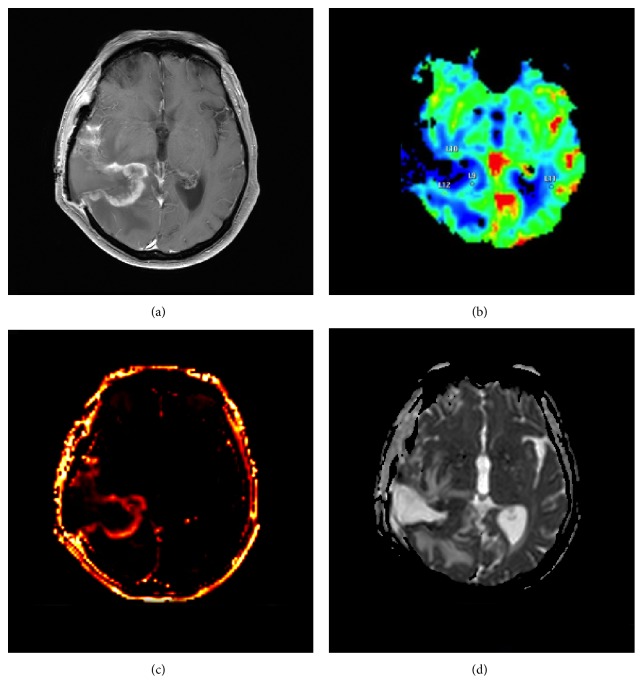
Enhancing lesion (a) during immunotherapy with dendritic cell vaccine. Mismatch between T1-enhancing volume and CBV (b), the last being just slightly elevated; permeability (*K*_trans_) is increased (c). ADC is low (d), suggesting hypercellularity.

Mixed scenarios with coexistence of glioma and treatment alterations are often the rule; moreover, with regard to the diagnostic specificity of contrast enhancement and of aMRI features, the situation after multimodal treatment could become confusing. It seems plausible that aMRI may provide deeper insights than cMRI in the recognition of pseudo or real glioma progression. On PWI, contrast-enhancing areas secondary to immunotherapy inflammation should be less perfused than progressive/recurrent tumor, but also, vessel disruption and thrombosis due to high malignancy may inversely affect the perfusional pattern. Nevertheless, inflammation increases vessel permeability with effects on perfusional parameters. Likewise, low ADC can be associated to both tumoral and immune hypercellularity, and specific analysis has to be performed to discriminate. MRS is useful to obtain metabolic information within the enhanced areas, by determining high choline concentration and therefore identifying glioma within treatment alterations (Figure 3). Particular interest in pseudoprogression is focused on the mismatch between CBV and enhancing volumes, permeability parameters, ADC analysis (Figure 4), and presence of lipids as possible substrate of NK cells.

In clinical practice, a combination of different techniques may be necessary to differentiate between pseudoprogression and tumor progression. Cut-offs in a single-shot examination cannot distinguish between progression and pseudoprogression, and the evaluation of longitudinal modifications of parameters in terms of intensity and pattern is recommended. MRI data need to be analyzed taking into account that gliomas are generally composed of different structural and functional regions and that multimodal treatments increase brain tissue heterogeneity. Two are the main approaches used: (a) the histogram approach [51, 59, 65, 89, 93, 122–125] in which the evolution of the tumor during therapy is completely and quantitatively characterized estimating the statistical parameters (number of peaks, kurtosis, skewness, and other statistical moments) of the distribution of the values inside the lesion and (b) the voxel-based approach [49, 50, 75, 126], which aims to detect predictive markers of therapeutic efficacy estimating voxel by voxel the difference between the parametric maps (mainly ADC, CBV, and *K*_trans_) of two different temporal points [75] [49]. An excellent example is that recently provided by Qin et al. in 2017 [98] based on an intermediate ADC (IADC) calculation in FLAIR VOI. In fact, the technique was able to discriminate patients with clinical benefit.

Imaging approaches like these, evolving in-depth analyses of MRI data that take into account whole-lesion heterogeneity and parametric modifications in the course of treatment such as parametric maps, TRAMs, and histogram analyses, deserve further investigation as they may provide the robust tools that are presently missing for the definition of PFS (i.e., progression-free survival) and clinical benefit in glioma immunotherapy.

## Figures and Tables

**Table 1:** iRANO criteria (modified from [14]).

RANO criteria for high-grade gliomas		
Complete response (CR)	(i) Disappearance of all enhancing disease for ≥4 weeks AND (ii) No new lesions AND (iii) Stable/improved T2/FLAIR AND (iv) No more than physiologic steroids AND (v) Stable/improved clinically	

Partial response (PR)	(i) ≥50% ↓ sum of biperpendicular diameters of enhancing disease for ≥4 weeks AND (ii) No new lesions AND (iii) Stable/improved T2/FLAIR AND (iv) Stable/improved steroids AND (v) Stable/improved clinically	

Stable disease (SD)	(i) Does not qualify for CR, PR, and PD AND (ii) No new lesions AND (iii) Stable/improved T2/FLAIR AND (iv) Stable/improved steroids AND (v) Stable/improved clinically	

Progressive disease (PD)	(i) ≥25% ↑ sum of biperpendicular diameters of enhancing disease OR (ii) New lesions OR (iii) Significant worsened T2/FLAIR OR (iv) Significant clinical decline	

iRANO criteria		

	If ≤6 months after start of IT	If >6 months after start of IT

Is a repeat scan required to confirm radiographic PD for patients without significant clinical decline?	Yes	No

Minimal time interval for confirmation of progression for patients without significant clinical decline	≥3 months	Not applicable

Is further immunotherapy (IT) treatment allowed after initial radiographic PD (if clinically stable) pending progression confirmation?	Yes	Not applicable

Does a new lesion define PD?	No	Yes

## Abbreviations

AD: Axial diffusivity
ADC: Apparent diffusion coefficient
aMRI: Advanced magnetic resonance imaging
ASL: Arterial spin labeling
BBB: Blood-brain barrier
CBF: Cerebral blood flow
CBV: Cerebral blood volume
Cho: Choline
cMRI: Conventional magnetic resonance imaging
CNS: Central nervous system
Cr: Creatine
DCE: Dynamic contrast enhanced
DSC: Dynamic susceptibility contrast
DTI: Diffusion tensor imaging
DWI: Diffusion-weighted imaging
FA: Fractional anisotropy
fDM: Functional diffusion maps
GBM: Glioblastoma multiforme
iAUC: Initial area under the curve
iRANO: Immunotherapy response assessment in neurooncology
ITSS: Intratumoral susceptibility signals
MD: Mean diffusivity
MRI: Magnetic resonance imaging
MRS: Magnetic resonance spectroscopy
NAA: N-acetyl aspartate
OS: Overall survival
PFS: Progression-free survival
PRM: Parametric response maps
RANO: Response assessment in neurooncology
RD: Radial diffusivity
ROI: Region of interest
SWI: Susceptibility-weighted imaging
TRAMs: Treatment response assessment maps
Ve: Extracellular extravascular volume fraction
VOI: Volume of interest.
